# Prevalence and Risk Factors of Intestinal Parasites in Cats from China

**DOI:** 10.1155/2015/967238

**Published:** 2015-05-11

**Authors:** Yurong Yang, Hongde Liang

**Affiliations:** Laboratory of Veterinary Pathology, College of Animal Science and Veterinary Medicine, Henan Agriculture University, Zhengzhou 450002, China

## Abstract

The prevalence of intestinal parasites in cats from China was largely unknown prior to this study. The aim of the present study was to investigate the presence of intestinal parasites in cats from central China and also identify risk factors for parasitism. Fecal samples from 360 cats were examined using sugar flotation procedure and fecal smear test by microscope. Cats had mixed two or three kinds of parasites infections. Of the 360 cats feces, intestinal parasites positive feces were 149 (41.39%). 64 (17.78%) were infected with *Toxocara cati*, 61 (16.94%) with *Isospora felis*, 41 (11.39%) with *Isospora rivolta*, 33 (9.17%) with *Paragonimus*, 23 (6.39%) with hookworms, 11 (3.06%) with *Toxoplasma*-like oocysts, 10 (2.78%) with *Trichuris*, 4 (1.11%) with lungworm, 2 (0.56%) with *Sarcocystis*, and 1 (0.28%) with *Trematode*. The cats' living outdoor was identified as risk factor by statistical analysis. These results provide relevant basic data for assessing the infection of intestinal parasites in cats from central region of China. In conclusion, there was high prevalence of intestinal parasites in cats from China.

## 1. Introduction

Gastrointestinal parasitism is one of the main causes of morbidity in domestic cats. The prevalence of intestinal parasites was varied due to geographical region, presence and frequency of veterinary care, season of the year, and the type of population of cat (stray, feral, shelter, household). The number of domestic cats in China (53 million) is quarter of the world (http://www.mapsofworld.com/world-top-ten/countries-with-most-pet-cat-population.html). But, little information on the prevalence of intestinal parasites in cats is available.

Here, we report the prevalence of intestinal parasites in cats from central China. This information is essential to veterinarians for the development of strategies for treatment and control of parasites and for public health authorities concerned with monitoring the zoonotic potential of infections in cats. The purpose of this study was to determine the regional prevalence of intestinal parasites and also identify risk factors for parasitism.

## 2. Materials and Methods

### 2.1. Cat Feces Study Area and Sampling

The survey was carried out in the central of China. The cat feces were collected from two major parts: Henan and Beijing. Henan (Latitude 34.90°N, Longitude 113.50°E) has a humid warm-temperate climate. The climate in Beijing (Latitude 39.54°N, Longitude 116.23°E) belongs to the warm temperate zone, half moist continental monsoon climate. The samples from Beijing (61 feces) came from one cat shelter. The samples from Henan came from stray cats (32 feces), market cats (205 feces), and household cats (62 feces). Total 360 cats fecal samples were collected by Veterinary pathology laboratory, Henan Agriculture University from 2013~2014.

### 2.2. Fecal Examination by Microscopy

Samples were examined by a conventional flotation method using 530 g/L sugar (specific gravity 1.15) as previously described [1]. Floating material was transferred to a slide and examined by light microscopy. The parasite eggs were differentiated according to their morphologic characteristics.

### 2.3. Statistical Analysis

The prevalence and frequency distribution of overall infection and of each parasite were tested by Graphpad software using Chi-square test. The analysis was performed considering the following independent variables: age (≤12 months old, >12 months old), management (outdoor or household), and habitation (Beijing or Henan). In statistical tests, *P* < 0.05 was regarded as significant.

### 2.4. Ethics

All investigations reported here were approved by the institutional animal use protocol committee of the Henan Agriculture University, China.

## 3. Results

The overall prevalence of intestinal parasites was 41.39% in cats (Table 1). The cats frequently mix infected two parasite species or three parasite species.* Toxocara cati* was the most prevalent species detected (17.78%) and then were* Isospora felis* (16.94%),* Isospora rivolta* (11.39%),* Paragonimus* (9.17%), hookworms (6.39%),* Toxoplasma*-like oocysts (3.06%),* Trichuris* (2.78%), lungworm (1.11%),* Sarcocystis* (0.56%), and* Trematode* (0.28%). The predominant predictors of intestinal parasite infection were habitation and housing. The infection rate of older cats and younger cats had no significant difference (OR = 1.01). Housing had impressive effect on the infection; outdoor cats showed higher infection risk than household cats (OR = 2.66). Cats from Beijing area showed less exposure to infections than cats from Henan area (OR = 13.42) (Table 2).

**Figure 1:**
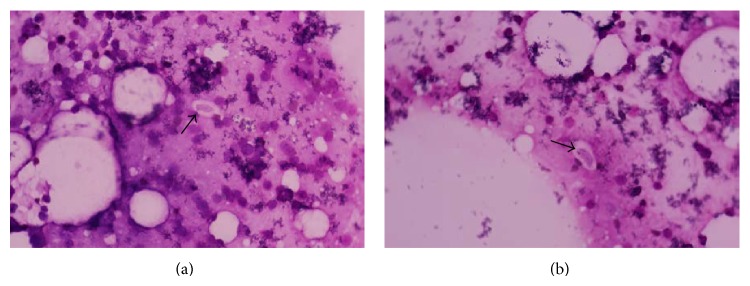
Results from smear revealed* Isospora felis* infection from the cat feces. Giemsa's stain ×1000. Two figures are photomicrographs of the cyst of* Isospora felis* (arrow). The IFN-*γ* knockout mice were inoculated cat fecal floats orally. The mice were killed at 6 weeks postinfection. The smears were from mesenteric lymph nodes of IFN-*γ* knockout mice.

All fecal floats were bioassayed in mice for another project (isolation* T. gondii*) irrespective of microscopic examination results. For mice bioassay, fecal floats were inoculated into SW and IFN-*γ* knockout mice in pools. 1 viable* T. gondii* was found from the cat feces.* Isospora felis* cysts were found in smear of the mesenteric lymph nodes of IFN-*γ* knockout mice 6 weeks postinfection (Figure 1). The size of sheath or cyst-like structures was 12.3 × 4.2 *μ*m, not including sheath, and 19.4 × 12.2 *μ*m with the sheath in smear. The* Isospora felis* cysts positive samples are all from Henan. But, we do not further check which sample and how many samples were positive for* Isospora felis*. From the muscle of IFN-*γ* knockout mice, the cysts of* Hammondia hammondi* were not found.

## 4. Discussion

This survey found the most common protozoa and helminth infections in cats were* Toxocara cati*,* Isospora felis*,* Isospora rivolta, Paragonimus*, hookworm, and* Trichuris* (Table 1). This represents the first comprehensive intestine parasites survey of cats from China. The investigation of the prevalence of gastrointestinal parasites in cats had been done in several countries. It was 8.6%~35% in the world [2–8]. In our study, the overall prevalence of intestinal parasites in cats was 41.39%; the prevalence of intestine parasites in cats from China was higher than other places' cat population. The different locations or housing had more difference: 48.49% in Henan province and 6.56% in Beijing; 28.48% in household cats and 51.49% in outdoor cats.

Toxocariasis is a widespread zoonosis caused by the* Toxocara canis* and* Toxocara cati*, which primarily infect dogs and cats, respectively.* Toxocara cati* could infect human and induce disease. Compared with another study in China, the epidemiological study of toxocariasis showed a seroprevalence of 12.25% (351/2866) in human [9]. Compared with a number of other studies (3.2%~22.2%) [3–5],* Toxocara cati* were found at a higher infection rate (17.78%) in our survey. Hookworm parasites were common helminth in cat; the prevalence in this study is 6.39%. This is lower than previous reports in other parts of China, and the infection rate of hookworms in cats from Sichuan and Guangdong was 25%~95% by necropsy or PCR [10]. Hookworms may cause zoonotic disease. Feline hookworm* Ancylostoma ceylanicum* larval infection in human could lead to hookworm associated cutaneous larva migrans. 2.78% (10/360)* Trichuris* eggs were found in this study. Human* Trichuris Trichiura*, pig* Trichuris suis*, and dog* Trichuris vulpis* could cause diarrhea and inflammation of the cecum and colon [11]. There are two species of whipworms (*Trichuris serrata, Trichuris campanula*) that can infect cats. But, to date there are no studies documenting clinical signs or pathology of them. The zoonotic potential of* Trichuris* in cats should be assessed further. 1.11% (4/360) cats were infected with lungworms. Lungworms could cause respiratory problems in cats. The most common types of worm to affect cats are* Aelurostrongylus abstrusus* and* Capillaria aerophila*. Prevalence rates of lungworms in cats from China are rare. Global prevalence rates vary from 50% in cats from Albania to 1% in cats from Spain [12].

*Paragonimus* has been reported infecting animals and humans, which have been identified in many parts of the world, including China [13]. The cats acting as a reservoir host of* Paragonimus*. Seroprevalence of* Paragonimus* in human from China ranges from 1.9% to 33.7% [14]. In cats, it was from 0.6%~44.7% in China [15, 16]. Compared to the prevalence of* Paragonimus* in 1980s in China, the prevalence of* Paragonimus* infection decreased in 2000s because of the disappearance of some wild animals and the shutdown of the life cycle. In this survey, the prevalence of* Paragonimus* in cat feces was 9.17%, all positive in Henan province.

We found a higher proportion of samples positive for* I. felis *(16.94%) and* Isospora rivolta* (11.39%) in this study from cat feces.* I. felis and I. rivolta* appear to be non pathogenic for cats [17]. The prevalence of* Isospora* in cats was 2.2%~9.0% in the world [6, 7, 18]. The survey about the prevalence of* Isospora* in China was rare and only in Chinese journal, it was 28.4%~33.0% in China [19, 20]. 3.06% (11/360)* Toxoplasma*-like oocysts were found in this study.* Toxoplasma*-like oocysts include* Toxoplasma gondii* and* H. hammondi*.* T. gondii* and* H. hammondi* were closely related tissue cyst forming coccidian parasites with a two-host life cycle.* T. gondii* causes infectious diseases in humans and animals [1], whereas no disease has yet been associated with* H. hammondi* [21]. Unlike* T. gondii*, which can be continuously passaged in mice, only the oocysts of* H. hammondi* are infective to mice and it cannot be maintained beyond the first mouse passage. The oocysts of both species (*T. gondii and H. hammondi*) were regarded as undistinguishable morphologically and serologically. The identification of the oocysts was accomplished by using molecular techniques or biological analysis in IFN-*γ* knockout mouse [22]. In natural environment,* T. gondii* oocysts were found in 1% of cats at any given time according to fecal surveys from 1988~2008 [1].

Our study is subject to limitations. First, only one fecal sample from each cat was collected and examined in the present study. Prepatent infections and the intermittent shedding of parasite stages may lead to underestimation of the prevalence of parasitic infection. Second, the cats feces from Beijing sample were all from one animal shelter. The results might not represent all the cat population in Beijing. It is better to collect sample consecutively from the same cat and collect different kinds of cats feces in the future survey.

## 5. Conclusion

The prevalence of intestinal parasites in cat from China was higher than in most cat population worldwide, especially* Toxocara cati*,* Isospora felis*,* Isospora rivolta*, and* Paragonimus*. The cat fecal information may represent a potential zoonotic risk of* Toxocara cati*, hookworm, and* Paragonimus* for human. Treatment and care should control these intestinal parasites in China. Risk groups like children and immunocompromised individuals should take special care during contact with cats. This study provided relevant basic data for veterinaries to develop strategies for treatment and control of parasites and for public health authorities concerned with monitoring the zoonotic potential of infections in cats.

## Figures and Tables

**Table 1:** Prevalence and confidence interval of intestinal parasites in 360 cat feces from China.

Parasites	Beijing	Henan	Total
	Number of positive results	Number of positive results	Number of positive results
	Prevalence %	Prevalence %	Prevalence %
	(95% CI)	(95% CI)	(95% CI)
*Toxocara cati *	1	63	64
	1.64	21.07	17.78
	(0.01–9.55)	(16.82–26.06)	(14.16–22.08)

*Isospora felis *	3	58	61
	4.92	19.40	16.94
	(1.14–14.03)	(15.30–24.27)	(13.41–21.18)

*Isospora rivolta *	1	40	41
	1.64	13.38	11.39
	(0.01–9.55)	(9.95–17.73)	(8.48–15.11)

Hookworms	1	22	23
	1.64	7.36	6.39
	(0.01–9.55)	(4.86–10.94)	(4.25–9.44)

*Paragonimus *	0	33	33
	—	11.04	9.17
	—	(7.93–15.13)	(6.57–12.62)

Trematode	1	0	1
	1.64	—	0.28
	(0.01–9.55)	—	(<0.01–1.72)

*Trichuris *	0	10	10
	—	3.34	2.78
	—	(1.75–6.13)	(1.45–5.11)

*Toxoplasma-*like	0	11	11
	—	3.68	3.06
	—	(1.99–6.54)	(1.65–5.45)

Lungworm	0	4	4
	—	1.34	1.11
	—	(0.40–3.51)	(0.33–2.93)

*Sarcocystis *	0	2	2
	—	0.67	0.56
	—	(0.02–2.57)	(0.02–2.14)

Overall prevalence	4/61	145/299	149
	6.56	48.49	41.39
	(2.12–16.14)	(42.89–54.14)	(6.42–46.54)

**Table 2:** Final multivariate analysis of risks factors associated with intestinal parasites in cats in China.

Variable	Risk factor	OR	95% CI	*P* value
Age	≤12 months	1.00		NA
	>12 months	1.01	0.55–1.84	0.97

Housing	Household	1.00		NA
	Outdoor	2.66	1.71–4.15	<0.0001

Habitation	Beijing	1.00		NA
	Henan	13.42	4.75–37.92	<0.0001

OR: odds ratio; (CI) 95%: confidence interval.
